# Prenatal nicotine sex-dependently alters adolescent dopamine system development

**DOI:** 10.1038/s41398-019-0640-1

**Published:** 2019-11-18

**Authors:** Jennifer B. Dwyer, Anjelica Cardenas, Ryan M. Franke, YiLing Chen, Yu Bai, James D. Belluzzi, Shahrdad Lotfipour, Frances M. Leslie

**Affiliations:** 1 Department of Pharmacology, University of California, 360 Med Surge II, Irvine, CA 92697 USA; 2 Department of Emergency Medicine, School of Medicine, University of California, Irvine, CA 92697 USA; 3 Department of Anatomy and Neurobiology, School of Medicine, University of California, Irvine, CA 92697 USA

**Keywords:** Neuroscience, Molecular neuroscience

## Abstract

Despite persistent public health initiatives, many women continue to smoke during pregnancy. Since maternal smoking has been linked to persisting sex-dependent neurobehavioral deficits in offspring, some consider nicotine to be a safer alternative to tobacco during pregnancy, and the use of electronic nicotine delivery systems is on the rise. We presently show, however, that sustained exposure to low doses of nicotine during fetal development, approximating plasma levels seen clinically with the nicotine patch, produces substantial changes in developing corticostriatal dopamine systems in adolescence. Briefly, pregnant dams were implanted on gestational day 4 with an osmotic minipump that delivered either saline (GS) or nicotine (3 mg/kg/day) (GN) for two weeks. At birth, pups were cross-fostered with treatment naïve dams and were handled daily. Biochemical analyses, signaling assays, and behavioral responses to cocaine were assessed on postnatal day 32, representative of adolescence in the rodent. GN treatment had both sex-dependent and sex-independent effects on prefrontal dopamine systems, altering Catechol-O-methyl transferase (COMT)-dependent dopamine turnover in males and norepinephrine transporter (NET) binding expression in both sexes. GN enhanced cocaine-induced locomotor activity in females, concomitant with GN-induced reductions in striatal dopamine transporter (DAT) binding. GN enhanced ventral striatal D2-like receptor expression and G-protein coupling, while altering the roles of D2 and D3 receptors in cocaine-induced behaviors. These data show that low-dose prenatal nicotine treatment sex-dependently alters corticostriatal dopamine system development, which may underlie clinical deficits seen in adolescents exposed to tobacco or nicotine in utero.

## Introduction

Many women continue to smoke tobacco during their pregnancies despite well-publicized risks to the developing offspring^1^. Maternal smoking (MS) has been linked to early-onset deficits in exposed infants, including low birth weight, increased risk of spontaneous abortion, neonatal withdrawal syndrome, sudden infant death syndrome, and difficulty arousing infants from sleep^2^. In addition to these early life deficits, MS is also linked to a set of delayed-onset neurobehavioral disorders that emerge in childhood and adolescence^3^. These include increased incidences of neuropsychiatric disorders like attention deficit-hyperactivity disorder (ADHD)^4–6^ and conduct disorder^7^, externalizing and aggressive behaviors^8,9^, low IQ^10,11^, and substance use disorders^4,12^. The risk of these neurobehavioral syndromes is influenced by sex, with males showing greater incidences of ADHD and conduct disorder, while females may be more at risk for substance abuse^4,13,14^. The etiologies of both ADHD^15,16^ and substance use disorders^17^ are thought to involve significant dysfunction of cortico-striatal-limbic circuits and their regulation by dopamine (DA). These mesocorticolimbic DA systems undergo substantial development during the adolescent period^18,19^, and may do so in sexually dimorphic ways^20,21^. We have suggested that in utero exposure to tobacco smoke targets late-maturing catecholamine systems, and that behavioral deficits relating to this exposure emerge only later in life as these circuits mature^2^. Support for this hypothesis has emerged from genetic studies suggesting that a polymorphism of the dopamine transporter (DAT) interacts with MS to further increase the risk of ADHD^22–24^, an interaction so potent that it is even observed with second-hand smoke^25^. It is not know whether these effects are mediated by tobacco exposure or by nicotine, the major psychoactive tobacco smoke constituent. Understanding the role played by nicotine is critical as the use electronic-cigarette nicotine delivery systems is on the rise, with up to 20% of adolescents reporting use in 2018^26^, and 5–9% of women reporting use during pregnancy^27,28^.

Animal models have been integral to understanding the mechanisms underlying MS effects. Most models have focused on nicotine, which can both activate and desensitize neuronal nicotinic acetylcholine receptors (nAChRs)^29^. nAChRs are present in human and rodent fetal brains^30–32^, and exhibit transient regional subunit and receptor expression during sensitive periods of brain development^2^. In rodents, gestational exposure to nicotine (GN) produces late-onset deficits in brain and behavior that largely parallel the delayed clinical onset of MS-related deficits. In particular, adolescent rats exposed to GN exhibit altered locomotor^33^, stereotypic^34^, and reward-related responses^34,35^ to indirect DA agonists such as cocaine, consistent with altered function of DA systems. While there is evidence that GN alters DA content in the forebrain^36–39^, no study has directly assessed sex differences in the effects of GN on developing dopamine systems in corticostriatolimbic circuitry during adolescence. Thus, this study tests the hypothesis that GN sex-dependently alters the organization and sensitivity of these systems during adolescence as measured by expression of monoamine transporters and DA receptors, D2-like G-protein coupling, tissue catecholamine content and turnover, and D2-like control of cocaine-induced behavior.

## Materials and methods

### Animals and tissue collection

Sprague–Dawley rats were maintained in a temperature-(21 °C) and humidity-(50%) controlled room on a 12 h light/dark cycle (lights on 07:00 h) with unlimited access to food and water. Pregnant rats (Charles River, USA) were treated with nicotine (Sigma, St, Louis, MO) or saline as previously described^40^. Each rat was given either nicotine at a concentration of 3 mg/kg/day (concentration expressed as base) or saline via an osmotic mini-pump (Alzet model 2002, flow rate 51 μl/day) from gestational days 4 to 18. After birth, litters were culled to ten and pups were cross-fostered to drug-naive mothers to minimize the effects of abnormal maternal rearing behaviors. Pups were weaned at postnatal day 21 (P21) and were group-housed in groups of 2–4 by sex. Litter was the experimental unit of analysis, and thus only one animal per litter was tested for each experimental measure. Radioligand binding and [^35^S]GTPγS studies drew from a total of 24 GS and 24 GN litters. Tissue catecholamine experiments drew from 30 GS and 30 GN litters, and behavioral studies drew from 30 GS and 30 GN litters. Please refer to the supplementary methods for more details on our approach. All experiments were performed in accordance with the Institutional Animal Care and Use Committee at the University of California, Irvine, and consistent with Federal guidelines.

### Radioligand binding

Brains from both males and females were cryostat sectioned at 20 μm thickness at −20 °C. Alternate sections from the same brain were cut for dopamine (DAT), norepinephrine (NET), and serotonin (SERT) transporter binding. Alternate sections from different animals were cut for D1, D2, and D3 receptor binding. Sections were thaw mounted onto poly L-lysine-coated slides, dehydrated at 4 °C for 2 h, and stored at −20 °C until use. DAT and SERT binding were measured using [^125^I] RTI-55, and NET binding was measured by [^3^H] nisoxetine, as previously described^41^. D1 binding was measured using [^3^H] SCH23390, D2 binding with [^125^I] Iodosulpiride, and D3 binding was measured by [^125^I]-7-OH-PIPAT^42^ (see Supplemental Methods for detail). Following incubation, slides were rinsed in ice-cold buffer and dipped in cold distilled water, then blown dry and exposed to Kodak Biomax film for 48 h with ^14^C standards of known radioactivity.

### G-protein coupling

A separate group of GS and GN males were taken directly from the homecage and sacrificed via rapid decapitation. Brains were removed and flash frozen in −20 °C isopentane, then stored for no more than 5 days at −70 °C. Quinelorane-stimulated [^35^S]GTPγS autoradiography was performed as previously described^42^. Twenty micrometer coronal sections were thaw-mounted onto Poly-L-lysine coated glass slides for the determination of basal and quinelorane-stimulated [^35^S]GTPγS binding, as well as nonspecific binding (see Supplemental Methods). Sections were apposed to Kodak MR films together with [^14^C] standards for 3 days.

### Quantitative analysis of autoradiograms

Autoradiographic images were quantified using a computer-based image analysis system (MCID, Image Research Inc., St Catharines, ON, Canada). Brain areas on autoradiograms were identified with reference to adjacent brain sections processed for cresyl violet stain^43^. Optical densities in discrete brain regions were measured and the corresponding values of radioactivity were determined by interpolation from a standard curve, generated from ^14^C standards of known radioactivity^30^. In each brain region, specific radioligand binding was quantified by subtracting corresponding regional measures of basal binding or nonspecific binding, for [^35^S]GTPγS or transporter and receptor binding, respectively. Radioligand binding was expressed as fmol/mg wet weight. Regional averages were obtained from readings of the right and left hemispheres from at least two comparable sections for each brain region. Regions of analysis are displayed in Table 1. Not all markers were expressed in all regions of interest. Areas that showed no expression were not analyzed.

**Table 1:** Analyzed brain regions

Region	Subregions
Prefrontal cortex (PFC)	Cingulate (Cg1)
	Prelimbic (PrL)
	Infralimbic (IL)
	Ventrolateral/orbital (VLO)
Striatum at rostral	Caudate putamen (CPu)-r
Striatum at middle	CPu-md
	CPu-mv
	CPu-mm
	CPu-mc
Nucleus accumbens	Core (NAcC)
	Shell (NAcSh)
Caudal levels	CPu-cm
	CPu-cv
	CPu-cd
	Cpu-cc
Islands of Calleja (ICj)	
Ventral pallidum (VP)	
Olfactory tubercle (Tu)	
Bed nucleus of the stria terminalis (BNST)	
Paraventricular nucleus of the hypothalamus (PVN)	
Amygdala	Basolateral nucleus (BLA)
	Central nucleus (CeA)
	Medial nucleus (MeA)
Regions containing dopaminergic cell bodies	Substantia nigra pars compacta (SNc)
	Reticulata (SNr)
	Ventral tegmental area (VTA)
	Locus coeruleus (LC)

### Tissue catecholamine levels

Brains collected from both males and females were dissected on an ice-chilled rat brain matrix (Plastics One, Roanoke, VA). One millimeter slices were taken that contained the medial prefrontal cortex (PFC), caudate putamen (CPu), nucleus accumbens (NAc), and the basolateral amygdala (BLA), which were identified with reference to a rat brain atlas^43^. Sections were quickly frozen on dry ice and tissue samples were dissected bilaterally using a 1 mm diameter tissue punch (Stoelting, Wood Dale, IL, USA, Integra, York, PA, USA). Punches were then expelled into 300 μl of ice-cold 0.1 M perchloric acid, and homogenized. Samples were centrifuged at 10,000×*g* for 10 min, and the resulting pellets were resuspended in 100 μl of 0.1 M NaOH before measuring the protein content using a Bradford protein assay kit (Bio-Rad, Hercules, CA). The supernatants were frozen at −70 °C until their use for the measurement of catecholamines and their metabolites using HPLC-ED.

### Cocaine-induced locomotor behavior

All behavioral testing was conducted using four identical open-field activity systems (Med Associates, St. Albans, VT) measuring 43.2 × 43.2 × 30.5 cm. Sixteen evenly spaced infrared monitors located on two adjacent sides of the chamber recorded horizontal locomotion. Parameters determining ambulatory activity were adjusted for the size of adolescent animals, using an infrared box size of 4. On test days, GS and GN rats were placed into the novel locomotor apparatus for a 10-min habituation period. In the first behavioral experiment, following habituation, rats were injected with saline or the D2-like antagonist haloperidol (0.1 mg/kg, i.p.) (Sigma-Aldrich). In the second experiment, following habituation, rats were injected with vehicle (100% ethanol) or the D2 selective antagonist L-741,626 (0.1 mg/kg, 1.0 mg/kg, 5.0 mg/kg, i.p.) (Sigma-Aldrich). In both behavioral experiments, all animals received an injection of cocaine (15 mg/kg) (National Institute of Drug Abuse) 20 min following the first injection, and locomotor behavior was recorded via computer-assisted data acquisition for 30 min. All drugs were dissolved in physiological saline, except L-741,626, which was dissolved in 100% ethanol.

### Data analysis

#### Transporter and receptor radioligand binding

Each transporter and receptor was analyzed separately via 3-way ANOVA for sex × gestational group × brain region as a repeated measure (SPSS 24.0, Chicago, IL). If there was a significant effect or interaction with brain region, subregions were analyzed separately. Each region was individually analyzed via 2-way ANOVA for sex × gestational group. If the effect of sex, or its interaction with gestational group was significant, then GN effects were analyzed separately in males and females using a two-tailed Student’s *t*-test, with Bonferonni correction to account for the multiple comparisons (three comparisons: GS male versus GS female, GS male versus GN male, and GS female versus GN female). If there was no effect or interaction with sex, males and females were pooled and gestational groups were compared via Student’s *t*-test.

#### [^35^S]GTPγS binding

Quinelorane-stimulated [^35^S]GTPγS binding was calculated as the percent increase in optical density relative to basal levels in each brain region (i.e., ((Quinelorane − Basal)/Basal) × 100). Quinelorane-stimulated [^35^S]GTPγS binding was analyzed via a two-way ANOVA with repeated measures, with brain region as the within factor and gestational group as the between factor. If there was a significant within effect or interaction with gestational group, GN-induced differences in each area were assessed separately. GS and GN quinelorane-stimulated [^35^S]GTPγS binding were compared using Student’s *t*-tests. Data from all autoradiography experiments were expressed as the mean ± SEM of each experimental group.

#### Tissue catecholamine content

Each catecholamine, metabolite, and metabolite ratio was analyzed separately in each brain region. Data were analyzed via 2-way ANOVA with sex × gestational group as dependent variables. If there were significant effects of sex of interactions of sex with gestational group, males and females were analyzed separately. Differences between GS and GN animals were determined via Student’s *t*-test with Bonferroni correction for multiple comparisons (three comparisons: GS male versus GS female, GS male versus GN male, and GS female versus GN female).

#### Locomotor behavior

In experiment 1, cocaine-stimulated ambulatory activity was analyzed by 3-way ANOVA for sex × gestational treatment × antagonist pretreatment, with Bonferroni adjustment for multiple comparisons (three comparisons: GS versus GN, GS saline versus haloperidol, and GN saline versus haloperidol. In experiment 2, cocaine-stimulated ambulatory activity was analyzed via 2-way ANOVA for gestational treatment × antagonist dose. All statistically significant effects or interactions were further analyzed via one-way ANOVAs with Bonferroni-adjusted post hoc comparisons comparing all four drug doses to each other (vehicle, 0.1 mg/kg, 1 mg/kg, and 5 mg/kg). Differences were considered statistically significant at *p* < 0.05.

## Results

### Prefrontal cortex

**Fig. 1:**
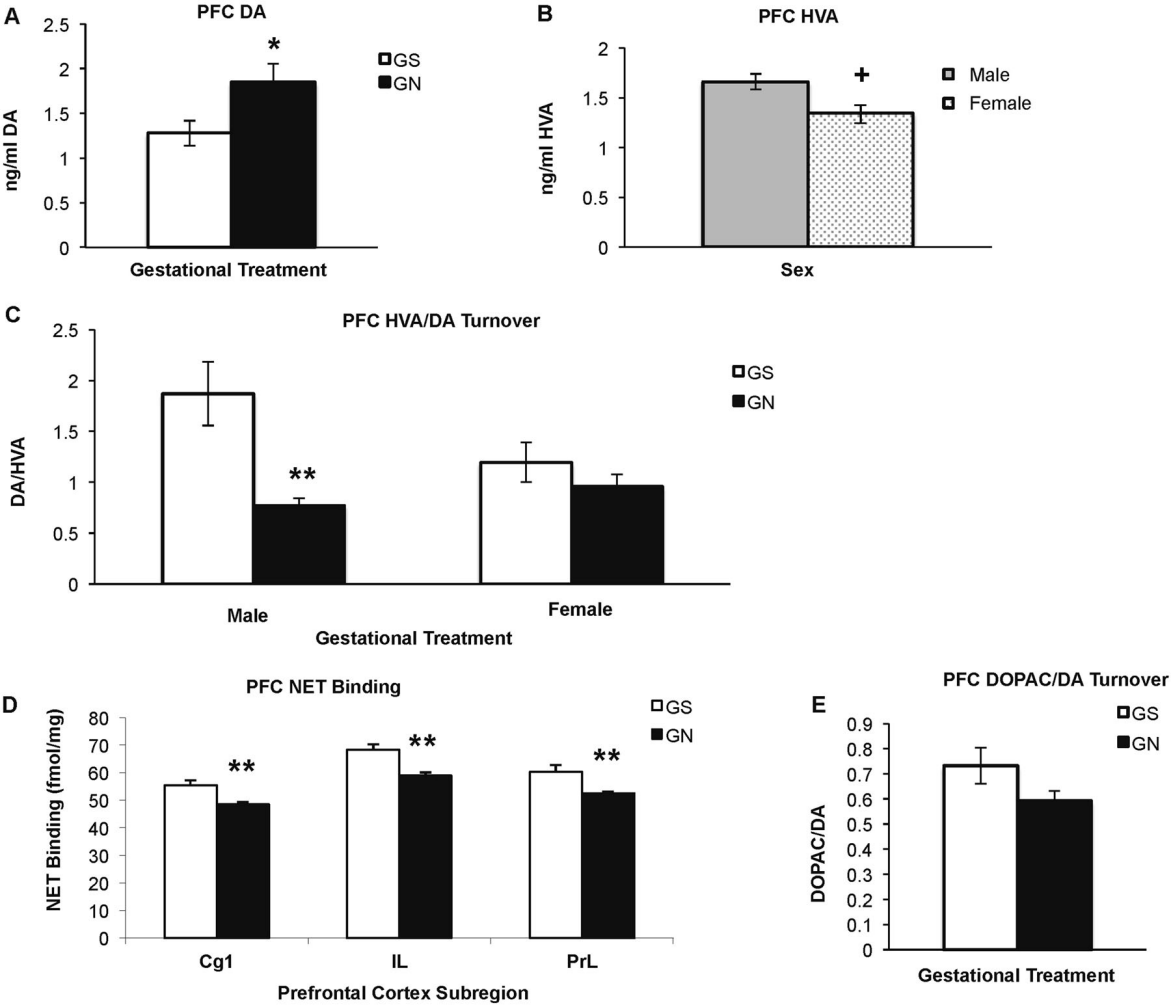
GN alters dopamine content, metabolism, and NET expression in the prefrontal cortex. **a** GN increases tissue dopamine content in male and female adolescents. **b** HVA content is not altered by GN. **c** GN decreases COMT-dependent dopamine turnover to HVA in male, but not female adolescents. *p < 0.05 GS versus GN, **p < 0.01, ^+^*p* < 0.05 males versus females *n* = 9–12 per group. **d** Regardless of sex, GN decreases NET binding in the medial prefrontal cortex. **e** There was a trend towards decreased transporter-dependent dopamine turnover to DOPAC in GN-treated animals in the prefrontal cortex. **p < 0.01 GS versus GN; *n* = 8 GS and 8 GN per group (NET binding) and *n* = 9–11 per group (DOPAC/DA).

Tissue levels of DA and its metabolites were assessed in the PFC of male and female GS and GN adolescents (Fig. 1). PFC DA was significantly regulated by GN (Fig. 1a) (F(1,40) = 4.710, *p* = 0.036), but not by sex (F(1,40) = 0.259, *p* = 0.61) nor its interaction with GN (F(1,40) = 0.453, *p* = 0.505), with increased PFC DA content in GN treated animals. Although the DA metabolite, homovanillic acid (HVA) was higher in males than in females (F(1,42) = 6.069, *p* = 0.018, Fig. 1b), it was not altered by GN (F(1,42) = 0.250, *p* = 0.62) nor its interaction with sex (F(1,42) = 0.077, *p* = 0.78). The turnover ratio of HVA to DA (Fig. 1c) was significantly influenced by GN (F(1,36) = 11.385, *p* = 0.002), and by the interaction of GN with sex (F(1,36) = 4.725, *p* = 0.036), but not sex alone (F(1,36) = 1.538, *p* = 0.22). GN reduced DA turnover to HVA in males (*p* = 0.009), but not females.

Although GN sex-dependently altered Catechol-O-methyl transferase (COMT)-dependent DA turnover to HVA, monoamine transporter expression was altered in both sexes. As previously reported^44^, DAT binding was low in the PFC, and was not influenced by sex or GN (Supplemental Results). In contrast, PFC NET binding (Fig. 1d) showed more robust expression and was significantly decreased by GN treatment (F(1,12 = 12.470, *p* = 0.004) regardless of sex (sex: F(1,12) = 0.623, *p* = 0.45; interaction: F(1,12) = 0.114, *p* = 0.74). The medial PFC showed a significant effect of subregion (F(3,36) = 93.149, *p* < 0.001); however when analyzed separately, the Cg1, IL, PrL and VLO subregions all showed reduced NET binding in GN animals (Fig. 1d, Supplemental Results). Consistent with NET’s DA uptake function in the PFC^45^, there was a trend towards GN regulation of transporter-dependent DA turnover to 3,4-Dihydroxyphenylacetic acid (DOPAC) (Fig. 1e). The turnover ratio of DOPAC to DA showed no effect of sex (F(1,36) = 0.600, *p* = 0.443) nor a sex by GN interaction (F(1,36) = 1.423, *p* *=* 0.241), but there was a trend towards GN regulation (F(1,36) = 2.423, *p* = 0.128). Although there were no significant GN-related differences in SERT or D1 receptor binding (Supplemental Results), there was a trend of increased quinelorane-stimulated [^35^S]GTPγS in GN-treated males in the PFC (F(1,10) = 3.656, *p* = 0.085) (data not shown), suggesting that D2 functional coupling may be enhanced in this region.

### BLA

GN did not influence transporter binding, DA receptor binding, quinelorane-stimulated [^35^S]GTPγS, tissue DA content, or turnover in the BLA (Supplemental Results). Tissue norepinephrine (NE) content was significantly influenced by sex (F(1,49) = 4.226, *p* = 0.045) with higher NE content in females compared to males by nearly 60%, but there were no significant influences of GN (F(1,49) = 0.209, *p* = 0.650) nor its interaction with sex (F(1,49) = 0.761, *p* = 0.387) (data not shown).

### Striatum

**Fig. 2:**
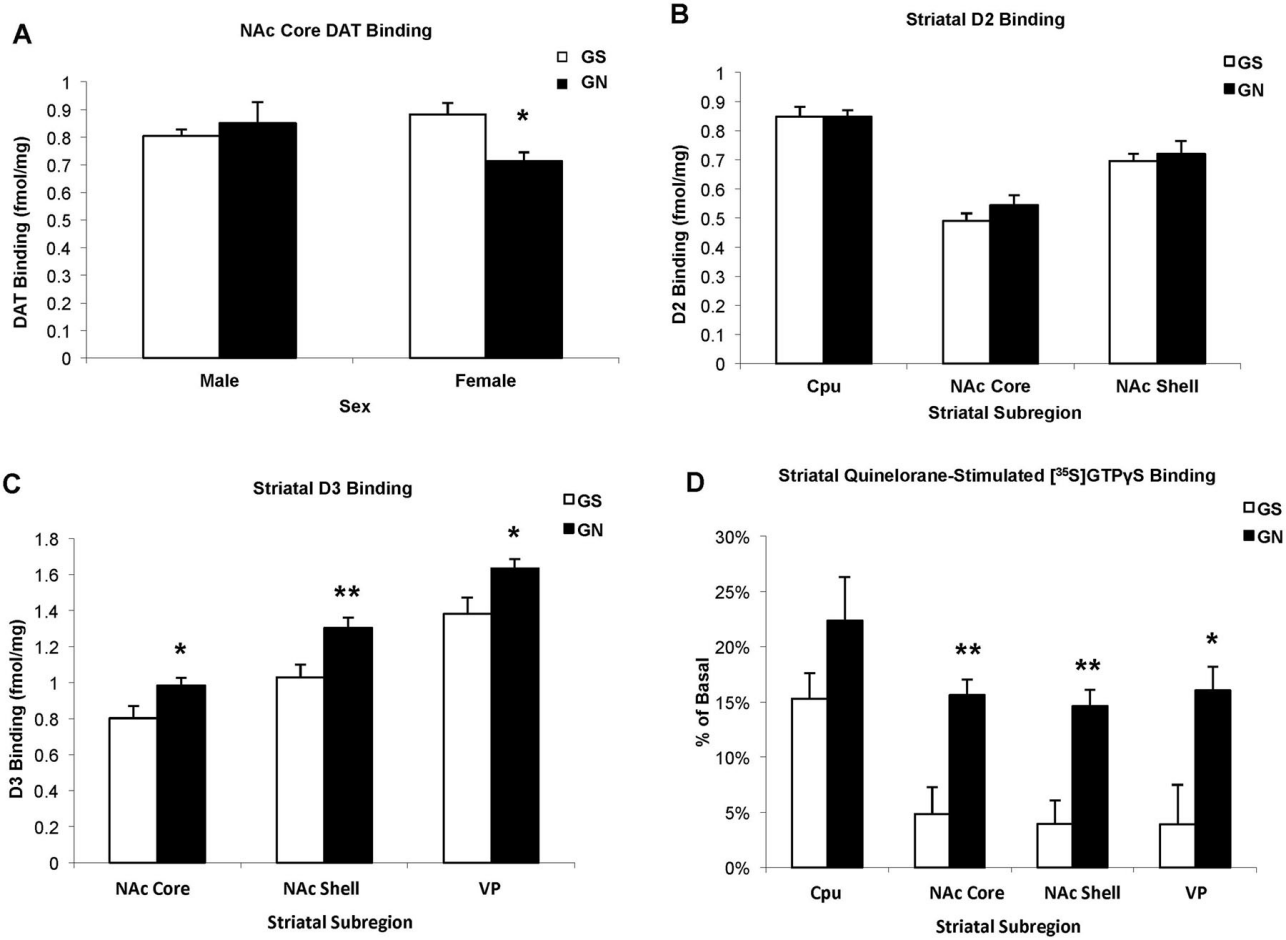
GN sex-dependently alters striatal DAT binding and alters D2-like binding and functional coupling. **a** GN decreased DAT binding in the NAc core of females, but not males. *p < 0.05 GS versus GN of same sex; *n* = 4–10 per group. **b** GN does not alter striatal D2 binding in male or female adolescents (because there were no sex differences, males and females were pooled). **c** GN increases striatal and pallidal D3 binding regardless of sex. **d** GN treatment increases D2 functional coupling in the ventral striatum and pallidum. *p < 0.05, **p < 0.01 GS versus GN; *n* = 14–16 per group (D2 and D3 binding; males and females pooled); *n* = 6 per group ([^35^S]GTPγS binding in males only).

While DAT binding in the dorsal striatum and NAc shell did not show significant treatment differences (Supplemental Results), DAT binding in the NAc core (Fig. 2a) was significantly influenced by sex (F(1,21) = 4.268, *p* = 0.051) and the interaction of sex with GN (F(1,21) = 4.964, *p* = 0.034), but not by GN alone (F(1,21) = 1.529, *p* = 0.23). GN significantly decreased DAT binding in females (*p* = 0.022), but not males. There were no significant differences in NET or SERT binding due to GN or sex (Supplemental Results), suggesting that GN effects in this region are selective for the DA system.

GN treatment did not significantly alter D1 (Supplemental Results) or D2 binding (Fig. 2b) in dorsal striatal (GN: F(1,24) = 0.014, *p* = 0.91), rostral NAc core (GN: F(1,28) = 0.823, *p* = 0.37) and shell (GN: F(1,26) = 1.52, *p* = 0.23), or midbrain regions (ventral tegmental area GN: F(1,23) = 0.171, *p* = 0.67; substantia nigra GN: F(1,24) = 0.069, *p* = 0,80), although D2 binding was higher in females than in males in the centromedian CPu (F(1,26) = 7.945, *p* = 0.01) and the caudal segment of the NAc core (F(1,26) = 8.462, *p* = 0.007). In contrast, D3 binding (Fig. 2c) was significantly increased by GN treatment regardless of sex in the rostral NAc core (GN: F(1,23) = 5.517, *p* = 0.028; sex (F(1,23) = 0.290, *p* = 0.60; interaction F(1,23) = 0.540, *p* = 0.47), caudal NAc shell (GN: F(1,24) = 8.452, *p* = 0.008; sex: (F(1,24) = 1.238, *p* = 0.28; interaction: F(1,24) = 0.305, *p* = 0.59), and ventral pallidum (VP) (GN: F(1,24) = 6.020, *p* = 0.02; sex: (F(1,24) = 0.654, p = 0.43; interaction: F(1,24) = 1.825, *p* = 0.19). Since there were minimal sex differences in D2 or D3 binding, quinelorane-stimulated [^35^S]GTPγS (Fig. 2d) was examined in male animals, and was significantly influenced by striatal subregion (F(3,30) = 15.027, *p* < 0.001) and GN (F(1,10) = 11.175, *p* = 0.007). While there was only a trend in the dorsal striatum (e.g., Cpu, *p* = 0.153), GN significantly increased quinelorane-stimulated [^35^S]GTPγS binding in the NAc core (*p* = 0.003), NAc shell (*p* = 0.002), and the VP (*p* = 0.016). Despite the influence of GN on DAT binding and D2-like receptor properties, there were no effects of GN on overall catecholamine content or turnover (Supplemental Results).

### Cocaine-mediated behaviors

**Fig. 3:**
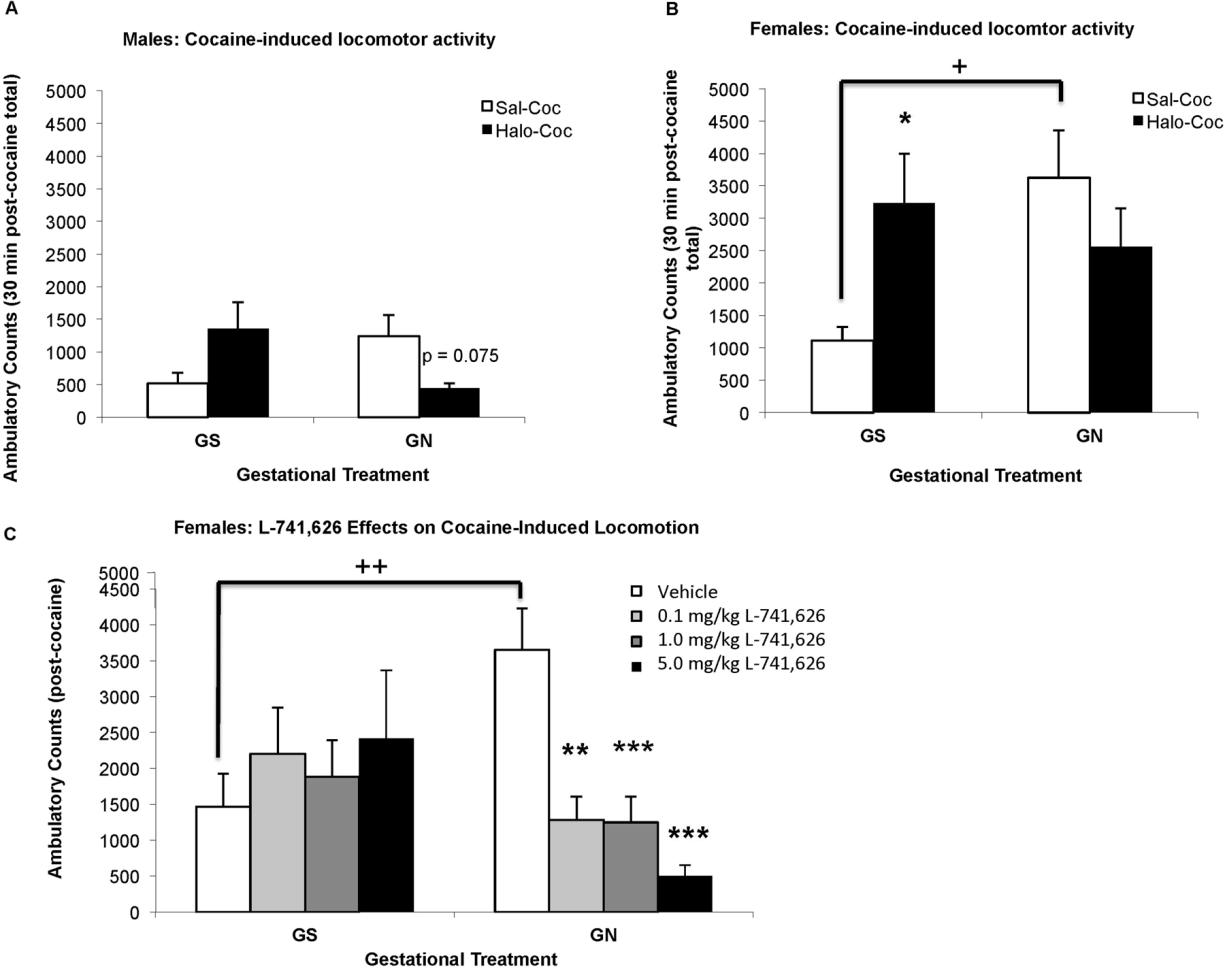
GN alters D2-like receptor control of cocaine-induced locomotion. **a** GN does not significantly alter cocaine-induced locomotion in adolescent males, although there is a strong trend for haloperidol decreasing cocaine-induced behavior in GN, but not GS, treated males. **b** GS females have decreased haloperidol-sensitive cocaine-induced locomotion compared to GN females, who are haloperidol insensitive. **c** GN females have greater L-741,626-sensitive cocaine-induced locomotion compared to GS females, who are L-741-626 insensitive ^+^*p* < 0.05 GN versus GS, ^++^*p* < 0.01; *p < 0.05, **p < 0.01 antagonist versus saline/vehicle within gestational group; panels **a** and **b** *n* = 12–16 per group; panel **c** *n* = 9–13 per group.

Since GN alters ventral striatal DAT binding in females, sex differences in cocaine-induced locomotor activity were assessed. Given the changes in D3 receptor expression and D2-like functional coupling, the roles of D2-like receptors were assessed using haloperidol. Cocaine-induced locomotion was significantly influenced by sex (F(1,105) = 23.21, *p* < 0.001), with higher locomotor activity in females (Fig. 3). While locomotion was not significantly influenced by GN (F(1,105) = 1.31, *p* = 0.255), there was an interaction of GN with haloperidol pretreatment (F(1,105) = 11.21, *p* = 0.001). When analyzed separately, male cocaine-induced locomotion (Fig. 3a) was significantly influenced by the interaction of GN with haloperidol pretreatment (F(1,49) = 8.495, *p* = 0.005), with a trend of reduced cocaine-induced locomotion following haloperidol treatment in GN but not GS males (*p* = 0.075). In females (Fig. 3b), cocaine-induced behavior was also significantly influenced by the interaction of GN with haloperidol pretreatment (F(1,56) = 6.397, *p* = 0.014). GN females, however, had significantly higher cocaine-induced locomotion compared to GS females (*p* = 0.015), and were insensitive to haloperidol pretreatment. In contrast, haloperidol pretreatment increased cocaine-induced locomotion in GS females (*p* = 0.039).

Given the more robust behavior in females relative to males, and the non-selectivity of haloperidol within the D2-like family, female animals were pretreated with the selective antagonist L-741,626^46^ prior to cocaine to examine the role of D2 receptors (Fig. 3c). Cocaine-induced locomotion was significantly influenced by the interaction of antagonist pretreatment with GN (F(3,79) = 5.441, *p* = 0.002). As observed in the first behavioral experiment, vehicle pretreated GN females had greater cocaine-induced locomotion compared to GS females (*p* = 0.006). When analyzed separately, there was no effect of L-741,626 pretreatment on cocaine-induced locomotion in GS females (F(3,40) = 0.431, *p* = 0.732). In contrast, GN females were sensitive to L-741,626 pretreatment (F(3,39) = 11.121, *p* < 0.001), with inhibition of cocaine induced locomotion at the 0.1 mg/kg (*p* = 0.002), 1.0 mg/kg (*p* = 0.001), and 5.0 mg/kg (*p* < 0.001) doses.

## Discussion

These data suggest that GN alters adolescent DA system development in corticostriatal circuits in both sex-dependent and sex-independent ways, largely consistent with gender differences described in the clinical MS literature. GN increased PFC DA in both sexes, but its COMT-dependent turnover to HVA^47^ was decreased only in males (Fig. 1). Both sexes exhibited a decline in NET transporter binding, with a trend towards decreased transporter-dependent turnover to DOPAC (Fig. 1c, d). Although males showed greater alterations in measures of prefrontal DA function, striatal DAT binding was selectively reduced in females, who also showed greater locomotor responses to cocaine. D1 receptor binding was insensitive to GN treatment, whereas D2-like receptors were more vulnerable. GN increased D3 binding in the ventral striatum and pallidum of both sexes, and increased quinelorane-stimulated [^35^S]GTPγS binding in the ventral striatum of males, although coupling was not assessed in females (Fig. 2). GN altered D2-like receptor control of cocaine-induced locomotion in which GS females were sensitive to haloperidol but not L-741,626, and GN females were sensitive to L-741,626 but not haloperidol, suggesting differing D2 and D3 mechanisms.

### GN alters corticolimbic DA system development

Despite measuring markers of DA, NE, and serotonin function in several brain regions, GN-associated changes were primarily found within the DA systems in corticostriatal areas. Corticolimbic circuits mature throughout the adolescent period^48^, and the present data suggest that DA content and turnover in these circuits are sensitive to GN treatment. GN increased PFC DA content in males and females, suggesting that prenatal nicotine exposure fundamentally alters dopaminergic development regardless of sex. In the PFC, DA is metabolized by a COMT-dependent extracellular pathway, as well as by a transporter-dependent and monoamine oxidase-dependent intracellular pathway^47^. Since DAT levels are very low in this region, NET is thought to provide the primary means of DA reuptake^45^. GN decreased NET levels in both males and females and there was a trend towards reduced NET-dependent metabolism in both sexes. However, GN reduced COMT-dependent turnover in males, but not females. Prior studies have reported a GN-induced decrease of DA to HVA turnover at P22 in the male forebrain^49^, but female animals were not studied. Thus, the present findings suggest that these effects are sex-dependent and that males may be more sensitive to GN-induced alterations in extracellular DA metabolism. Human genetic studies implicate altered prefrontal COMT function in the etiology of conduct disorder and ADHD^50^, disorders whose risk is increased by MS exposure, particularly in males^4^. The male-selective effect of GN on COMT-dependent turnover may serve as a link between early exposure to nicotine or tobacco smoke and subsequent ADHD.

### GN alters striatal dopamine system development

Dopaminergic markers in the dorsal and ventral striatum were also sensitive to GN treatment, although alterations were observed at the level of receptor expression and function, rather than in tissue catecholamine content, consistent with some^36,38^ but not all^37^ previous studies. GN treatment decreased DAT binding in the NAc Core in females but not males (Fig. 2a). Human genetic studies suggest that decreased DAT expression (9 repeat (low expression) versus 10 repeat (high expression) DAT allele) correlates with increased striatal reactivity to rewarding stimuli, which may enhance susceptibility to drug addiction^51^. Therefore, the selective GN-induced reduction of striatal DAT in females could relate to the enhanced vulnerability to substance abuse in women exposed to MS^13^. GN treatment has been shown to enhance cocaine intake in self-administration paradigms in male adolescent rats, but females were not assessed^34^. In this study, GN females showed increased locomotor activity in response to cocaine compared to GS females and both GS and GN treated males (Fig. 3). Future studies should examine sex differences in cocaine reward.

Although the effects of GN on DAT expression were sex-dependent, the effects of GN on D2 and D3 receptor binding were sex-independent. GN did not alter striatal D2 binding in males or females, but significantly increased D3 binding in the ventral striatum and pallidum. While the function of the D3 receptor is incompletely understood, it has been heavily implicated in reward circuitry and drug dependence^52^. For example, post-mortem analysis of human cocaine addicts reveals increased D3 expression in the nucleus accumbens^53,54^. However, animals treated chronically with cocaine also exhibit increased D3 expression^55^, suggesting that upregulation is a consequence of drug exposure, rather than a predisposing factor to drug seeking. Accumbens D3 expression is controlled by brain derived neurotrophic factor (BDNF) released from DA neurons, and upregulation of the BDNF-D3 pathway is thought to facilitate the development of behavioral sensitization^56^. Intriguingly, GN upregulates BDNF expression in the nucleus accumbens^57^, and GN animals exhibit behavioral sensitization to cocaine, while GS adolescents do not^33^. Thus, increased D3 expression in GN animals may occur downstream of alterations in growth factor expression, and may contribute to enhanced behavioral plasticity to cocaine in GN animals, a hypothesis requiring further testing.

### GN alters D2-like function

Because there were no sex differences in D2 and D3 receptor binding, D2-like functional coupling was assessed in males only. In male adolescents, GN increased quinelorane-stimulated [^35^S]GTPγS binding in the NAc Core, NAc Shell, and the VP, with a trend towards enhanced binding in the CPu. While quinelorane has affinity for D2, D3, and D4 receptors^58,59^, quinelorane-stimulated [^35^S]GTPγS binding in the rat striatum is thought to reflect D2 activation, as it is blocked by the D2 selective antagonist L-741,626^60^. However selective antagonists were not employed in this study, and thus the possibility of a contribution of D3 receptors cannot be ruled out. Regardless of the relative contributions of D2 and D3, however, these data are the first to show that GN alters signaling properties of D2-like receptors in DA terminal fields, suggesting that developmental nicotine exposure has long-lasting consequences on DA receptor function.

Given the changes in D3 binding sites and the alterations in D2-like functional coupling in the ventral striatal circuitry regulating locomotor activity^61–63^, the contributions of D2-like receptors to cocaine-induced locomotion were assessed. GS females showed enhancement of cocaine-induced locomotion following haloperidol and insensitivity to L-741,626, suggesting an inhibitory role for D3 in locomotor behaviors in normally-developing adolescents, consistent with its known roles in adults^64^. The cocaine-induced locomotion effects following haloperidol had similar trends in males. When looking at females, GN animals showed insensitivity to haloperidol, but reduced cocaine-induced locomotion following L-741,626 treatment, suggesting an integral role for D2 receptors. Whether similar effects would be observed in males to L-741,626 treatment is not known, and could be evaluated in future studies. Thus, GN females lack the D3 inhibitory mechanisms seen in GS animals, but express higher numbers of D3 binding sites in the ventral striatum. This upregulation could result from impaired downstream D3 signaling, and further study of D3 signaling mechanisms in GN-treated adolescents is warranted.

Taken together, these data show that prenatal nicotine treatment markedly and often sex-dependently alters adolescent DA system development, which is largely consistent with sex differences observed in MS-related deficits. These changes include altered COMT-dependent metabolism in males, consistent with their associations with conduct disorder and ADHD^50,65^, and alterations in striatal DAT expression in females, consistent with its purported link to altered reward sensitivity^51^. These data also implicate selective changes in D2-like receptor function, which warrant further exploration given the important role of D2 in developmental psychopathologies and its common use as a clinical drug target. It is critical to note that these alterations in neurochemistry and behavior are induced by brief treatment with moderate doses of nicotine. It has been increasingly suggested that nicotine replacement therapy, including electronic-cigarettes, may be a safe alternative to smoking in pregnancy, with some advocating higher nicotine replacement doses for pregnant women in order to compensate for placental metabolism^66^. These rodent studies argue against the safety of even moderate-dose nicotine exposure during pregnancy, and suggest that nicotine itself is a neuroteratogen, with important implications for prenatal health counseling.

## Supplementary information

Supplemental Methods and Results
