# Supplementary material for: Prenatal nicotine sex-dependently alters adolescent dopamine system development
Source: Transl Psychiatry. 2019 Nov 18;9:304. doi: 10.1038/s41398-019-0640-1 (PMC6861272; doi:10.1038/s41398-019-0640-1)
Supplement: Supplementary file 1 — Supplemental Methods and Results [file 41398_2019_640_MOESM1_ESM.docx]

**Animal and Tissue Collection:**

Blood nicotine concentrations are comparable to levels found in humans who smoke about 1.5 packs of cigarettes per day (1), approximately 15–45 ng/ml (2). As previously reported (3), GN treatment at this moderate dose did not influence dam weight gain, litter size, or pup weight gain during postnatal development. Pups were weaned at postnatal day 21 (P21) and group-housed by sex until day P32 or P33, when they were either tested behaviorally or sacrificed via rapid decapitation for biochemical studies. Sample sizes were selected based on prior experience with biochemical (4-7) and behavioral experiments with adolescent rodents (3, 8-11). Brains being used for radioligand binding were rapidly removed, flash frozen in -20°C isopentane, and stored at −70°C until use. For determination of regional catecholamine content, brains were freshly dissected. For data analysis (biochemical experiments and automated locomotor data), animals were assigned 5-digit numbers in order to blind processing and analysis from group assignments or treatment information.

**Tissue Catecholamine Levels:**

At the time of the assay, thawed brain punch supernatant was filtered twice through a 0.22 µm Millex syringe filter (Millipore, Billerica, MA). 20 µl of each filtered sample was automatically injected by an ESA 542 refrigerated autosampler onto a MD-150 x 3.2 mm ODS C18 column (ESA Inc., Chelmsford MA, Thermo Scientific) connected to an ESA 580 HPLC or Dionex UltiMate 3000 (Thermo Scientific) pump. The column was kept at 35°C and perfused by MD-TM mobile phase (ESA, Chelmsford, MA, Thermo Scientific) at a rate of 0.6 ml/min. DA, norepinephrine (NE), serotonin and their metabolite levels were determined by an electrochemical ESA 5600 CoulArray detector with an ESA 5020 guard cell, a 5014B or Dionex 5011A electrochemical detector with the dominant potential of 300 mV and 240 mV, respectively. The sensitivity of the detectors is 500 fg. Measurements were analyzed using CoulArray for Windows32 Software 2.0 (ESA Inc., Chelmsford, MA, USA). Standard curves were generated with catecholamine (ESA, Chelmsford, MA), DOPAC, and HVA (Sigma-Aldrich, St Louis, MO) standards, and levels in experimental samples were determined from the curve and expressed as pg per 20 µl. Punch catecholamine concentrations were normalized to protein content and expressed as ng catecholamine/mg of tissue.

**Dopamine Receptor Radioligand Binding**

Brains collected from GS and GN treated male and female adolescents (P32) were cryostat sectioned at 20 μm thickness at -20°C. Alternate sections from the same brain were cut for D1, D2, and D3 receptor binding. Sections were thaw mounted onto poly L-lysine-coated slides, dehydrated at 4°C for 2h, and stored at -20°C until use. D1 binding was measured using [^3^H] SCH23390 according to the method of Marshall et al.(12), D2 binding was assessed using [^125^I] Iodosulpiride according to the method of Harrod et al.(13), and D3 binding was measured by [^125^I]-7-OH-PIPAT according to the method of Harrod et al. (13). Following incubation, all sections were rinsed in ice-cold buffer for 20 min. After a brief rinse with cold distilled water, slides were blown dry, and exposed to Kodak Biomax film for 48h with ^14^C standards of known radioactivity.

**Neurotransmitter Outlier Analysis and Data Not Quantified:**

Neurotransmitter data were acquired in three batches using two separate detectors, 5014B detector (ESA Inc., Chelmsford, MA, USA), for batches 1 and 2, and additional animals were added using Dionex 5011A detector (Thermo Scientific) for batch 3). HPLC runs were counterbalanced for GS and GN groups and for males and females. Initial statistical analysis revealed a significant effect of run date, and thus a conversion factor was calculated to standardize batches 2 and 3 to batch 1. Outliers were identified by splitting groups by gestational treatment and sex, and animals with data points that were greater than two standard deviations from the mean were omitted from the analysis.

**Supplemental Results:**

**Prefrontal Cortex:**

PFC DA, HVA, HVA/DA ratio, and DOPAC/DA ratio are reported in the main text. PFC DOPAC showed trends of regulation by GN (F(1,41) = 2.985, p = 0.09) and sex (F(1,41) = 3.142, p = 0.08), but no interaction (F(1,41) = 0.689, p = 0.41). There were no significant effects on PFC NE (GN: F(1,44) = 0.173, p = 0.68; sex: F(1,44) = 2.225, p = 0.14; interaction: F(1,44) = 0.163, p = 0.69) or 5-HT (GN: F(1,43) = 0.913, p = 0.35; sex: F(1,43) = 0.011, p = 0.92; interaction: F(1,43) = 0.320, p = 0.58). There was a significant effect of sex on the 5-HT metabolite, 5-HIAA, (F(1,44) = 5.437, p = 0.02), but no effect of GN (F(1,44) = 1.929, p = 0.17) nor its interaction with sex (F(1,44) = 0.526, p = 0.47). There were no significant effects on 5-HIAA/5-HT turnover (GN: F(1,40) = 0.495, p = 0.49; sex: F(1,40) = 0.017, p = 0.90; interaction: F(1,40) = 0.464, p = 0.50).

DAT binding in PFC was not significantly influenced by GN (F(1,24) = 0.814, p = 0.38), sex (F(1,24) = 0.036, p = 0.85), nor their interaction (F(1,24) = 0.097, p = 0.76). PFC NET binding was significantly influenced by GN, as described in the main text. NET binding at the PFC subregion level was also characterized by significant GN regulation: ventrolateral orbital (VLO): GN: F(1,12) = 10.147, p = 0.008), sex F(1,12) = 0.994, p=0.34), interaction F(1,12) = 0.449, p=0.52); prelimbic (PrL): GN: F(1,12) = 8.696, p = 0.012), sex F(1,12) = 0.502, p=0.49), interaction F(1,12) = 0.004, p=0.95); cingulate (Cg1): GN: F(1,12) = 10.316, p = 0.007), sex F(1,12) = 0.076, p=0.79), interaction F(1,12) = 0.023, p=0.88); infralimbic (IL): GN: F(1,12) = 12.989, p = 0.004), sex F(1,12) = 0.708, p=0.42), interaction F(1,12) = 0.117, p=0.74). PFC SERT expression was not significantly influenced by GN (F(1,12) = 0.002, p = 0.97), sex F(1,12) = 0.180, p=0.68), nor their interaction F(1,12) = 0.104, p=0.75).

D1 receptor binding in the PFC showed an overall effect of sex (F(1,20) = 5.237, p = 0.03) and of PFC subregion (F(4,20) = 151.948, p <0.0001), but no significant interactions. When subregions were analyzed separately, there were significant effects for sex in Cg1 (F(1,20) = 6.246, p = 0.02) and a trend in IL (F(1,20) = 3.376, p=0.08), both of which reflected higher D1 binding levels in males compared to females. There were no significant effects of GN (F(1,20) = 0.105, p = 0.75) or its interaction with sex (F(1,20) = 0.623, p = 0.44) at the PFC level, or within any PFC subregion. D2 and D3 receptor binding was not assessed in PFC.

**BLA**

There were no significant effects on dopamine or serotonin systems as measured via tissue catecholamine content. Results are described here for dopamine systems: DA (GN: F(1,54) = 0.239, p = 0.63; sex: F(1,54) = 0.112, p = 0.74; interaction: F(1,54) = 0.279, p = 0.60); DOPAC (GN: F(1,51) = 0.014, p = 0.91; sex: F(1,51) = 0.224, p = 0.64; interaction: F(1,51) = 1.389, p = 0.24); HVA (GN: F(1,9) = 1.122, p = 0.32; sex: F(1,9) = 0.030, p = 0.87; interaction: F(1,9) = 0.974, p = 0.35); DOPAC/DA (GN: F(1,48) = 0.001, p = 0.98; sex: F(1,48) = 0.677 p = 0.42; interaction: F(1,48) = 0.265, p = 0.61); HVA/DA (F(1,9) = 0.005, p = 0.95; sex: F(1,9) = 0.000, p = 0.99; interaction: F(1,9) = 0.610, p = 0.46). Results are described here for the serotonin system: 5-HT (GN: F(1,50) = 0.317, p = 0.57; sex: F(1,50) = 1.079, p = 0.30; interaction: F(1,50) = 0.072, p = 0.79); 5-HIAA (GN: F(1,51) = 0.554, p = 0.46; sex: F(1,51) = 0.153, p = 0.70; interaction: F(1,51) = 0.018, p = 0.89); 5-HIAA/5-HT ratio (GN: F(1,45) = 0.553, p = 0.46; sex: F(1,45) = 0.037, p = 0.85; interaction: F(1,45) = 0.007, p = 0.93).

DAT binding was not measured in the BLA. NET binding in BLA was not significantly influenced by GN (F(1,12) = 1.782, p = 0.21), sex F(1,12) = 0.651, p = 0.44), nor their interaction F(1,12) = 0.476, p = 0.50). BLA SERT binding was similarly unaffected (GN (F(1,12) = 0.966, p = 0.35), sex F(1,12) = 0.962, p = 0.35), interaction F(1,12) = 0.005, p = 0.95).

D1 binding was not affected by GN (F(1,20) = 0.003, p = 0.96), sex F(1,20) = 0.387, p = 0.54), nor their interaction F(1,12) = 0.521, p = 0.48). D2 and D3 binding were not assessed in the BLA.

**Striatum**

**Dorsal Striatum**

There were no significant effects of GN, nor interactions with sex for tissue catecholamines in the dorsal striatum as detailed here: NE: (GN: F(1,41) = 0.213, p = 0.65; sex: F(1,41) = 1.697, p = 0.20; interaction: F(1,41) = 2.824, p = 0.10); DA(GN: F(1,42) = 0.310, p = 0.58; sex: F(1,42) = 0.367, p = 0.55; interaction: F(1,42) = 0.126, p = 0.72); DOPAC (GN: F(1,40) = 0.001, p = 0.98; sex: F(1,40) = 0.293, p = 0.59; interaction: F(1,40) = 0.835, p = 0.37); HVA (GN: F(1,41) = 0.090, p = 0.77; sex: F(1,41) = 0.469, p = 0.50; interaction: F(1,41) = 0.019, p = 0.89); DOPAC/DA (GN: F(1,39) = 0.250, p = 0.62; sex: F(1,39) = 0.255, p = 0.62; interaction: F(1,39) = 0.431, p = 0.52); 5-HT (GN: F(1,40) = 0.288, p = 0.59; sex: F(1,40) = 0.061, p = 0.81; interaction: F(1,40) = 0.010, p = 0.92); 5-HIAA (GN: F(1,43) = 0.078, p = 0.78; sex: F(1,43) = 2.632, p = 0.11; interaction: F(1,43) = 0.194, p = 0.66).

There were trends or significant effects of sex on select turnover ratios in dorsal striatum, with higher ratios in males compared to females: HVA/DA (GN: F(1,40) = 3.003, p = 0.09; sex: F(1,40) = 3.936, p = 0.054; interaction: F(1,40) = 0.034, p = 0.86); When analyzed by group, the overall effect did not reach significance (F(3,40) = 2.196, p = 0.10) and individual comparisons revealed a trend toward GSMs having higher ratios than GNFs (Bonferonni corrected p = 0.086).

5-HIAA/5-HT: (GN: F(1,37) = 0.378, p = 0.54; sex: F(1,37) = 5.800, p = 0.02; interaction: F(1,37) = 1.394, p = 0.25), with higher turnover ratios in males compared to females. When analyzed separately by group (F(3,37) = 2.785, p = 0.054), there were no significant differences between GS males and females (p = 1.0), GS versus GN males (p = 0.65), nor GS versus GN females (p = 1.0).

DAT binding in the dorsal striatum was not significantly influenced by GN (F(1,23) = 1.264, p = 0.27), sex (F(1,23) = 0.303, p = 0.59), nor their interaction (F(1,23) = 0.518, p = 0.48). Similarly, NET binding was not significantly influenced by GN (F(1,12) = 1.076, p = 0.32), sex (F(1,12) = 0.002, p = 0.97), nor their interaction (F(1,12) = 0.053, p = 0.82), nor was SERT binding (GN: F(1,12) = 0.048, p = 0.83), sex F(1,12) = 0.165, p = 0.69), interaction F(1,12) = 0.726, p = 0.41).

D1 binding was unaffected in the dorsal striatum (GN: F(1,20) = 0.380, p = 0.55), sex F(1,20) = 0.011, p = 0.92), interaction F(1,20) = 0.007, p = 0.93). D2 binding in the dorsal striatum was not significantly influenced by GN (F(1,24) = 0.014, p = 0.91), sex (F(1,24) = 2.923, p = 0.10), nor their interaction (F(1,24) = 0.272, p = 0.61), although subregions significantly differed from one another (F(8,192) = 35.783, p<0.001). Subregion specific analyses of rostral, medial, and caudal segments yielded similar findings as the regional analysis, save for a significant sex difference in the centromedian caudate (sex: (F(1,26) = 7.945, p = 0.01; GN: F(1,26) = 0.089, p = 0.77; interaction: F(1,26) = 0.019, p = 0.89). D3 binding was not examined in the dorsal striatum.

**Ventral Striatum**

There were no significant effects of GN, sex, nor the interaction of GN with sex in ventral striatal catecholamine content or turnover: NE: (GN: F(1,51) = 0.535, p = 0.47; sex: F(1,51) = 0.352, p = 0.56; interaction: F(1,51) = 1.684, p = 0.20); DA: (GN: F(1,53) = 0.694, p = 0.41; sex: F(1,53) = 0.790, p = 0.38; interaction: F(1,53) = 0.932, p = 0.34); DOPAC: (GN: F(1,54) = 0.003, p = 0.96; sex: F(1,54) = 2.134, p = 0.15; interaction: F(1,54) = 0.417, p = 0.52); HVA: (GN: F(1,9) = 0.027, p = 0.87; sex: F(1,9) = 0.440, p = 0.52; interaction: F(1,9) = 1.305, p = 0.28); DOPAC/DA: (GN: F(1,50) = 0.413, p = 0.52; sex: F(1,50) = 0.032, p = 0.86; interaction: F(1,50) = 0.366, p = 0.55); HVA/DA: (GN: F(1,9) = 0.931, p = 0.36; sex: F(1,9) = 0.319, p = 0.59; interaction: F(1,9) = 0.392, p = 0.55); 5-HT: (GN: F(1,52) = 0.921, p = 0.34; sex: F(1,52) = 0.600, p = 0.44; interaction: F(1,52) = 0.624, p = 0.43); 5-HIAA: (GN: F(1,53) = 0.044, p = 0.84; sex: F(1,53) = 0.001, p = 0.98; interaction: F(1,53) = 0.068, p = 0.80); 5-HIAA/5-HT: (GN: F(1,49) = 0.983, p = 0.33; sex: F(1,49) = 0.707, p = 0.41; interaction: F(1,49) = 0.604, p = 0.44)

DAT binding in the NAc shell was not influenced by GN (F(1,22) = 0.027, p = 0.87), sex (F(1,22) = 0.056, p = 0.82), nor the interaction of sex with GN (F(1,22) = 1.748, p = 0.20).

There were no significant effects on NAc core NET binding (GN: F(1,12) = 1.571, p = 0.23, sex: F(1,12) = 0.034, p = 0.86, interaction: F(1,12) = 0.273, p = 0.61) or NAc shell NET binding (GN: F(1,12) = 0.365, p = 0.56), sex F(1,12) = 0.006, p = 0.94), interaction F(1,12) = 0.103, p = 0.75). There were no significant findings for SERT expression in the NAc core (GN: F(1,12) = 0.006, p = 0.94), sex F(1,12) = 0.535, p = 0.48), interaction F(1,12) = 0.010, p = 0.92), nor the NAc shell (GN: F(1,12) = 0.001, p = 0.98), sex F(1,12) = 0.139, p = 0.72), interaction F(1,12) = 0.079, p = 0.78).

D1 binding was unaffected in the ventral striatum by GN (F(1,20) = 0.015, p = 0.94), sex (F(1,20) = 0.006, p = 0.94), nor the GN x sex interaction F(1,20) = 0.230, p = 0.64). Regional analysis of ventral striatal D2 binding showed an effect of sex (F(1,24) = 5.006, p = 0.035) and non-significant trends of effects of GN (F(1,24) = 3.678, p = 0.07) and an interaction of GN with sex (F(1,24) = 3.965, p = 0.06). There was a significant effect of ventral striatal subregion (F(3,72) = 30.580, p<0.001), so the four subregions (caudal and rostral levels of core and shell) were analyzed separately. All subregions except the caudal NAc lacked significant main effects effects: Rostral NAc core (GN: F(1,28) = 0.823, p=0.37; sex: F(1,28) = 0.000, p = 0.98; interaction: F(1,28) = 0.129, p = 0.72), Rostral NAc shell (GN: F(1,26) = 1.515, p=0.23; sex: F(1,26) = 0.720, p = 0.40; interaction: F(1,26) = 1.803, p = 0.191), Caudal shell (GN: F(1,26) = 1.841, p=0.19; sex: F(1,26) = 3.018, p = 0.09; interaction: F(1,26) = 1.753, p = 0.197). The caudal NAc core showed a significant effect of sex (F(1,26) = 8.462, p = 0.01), no effect of GN alone (F(1,26) = 0.174, p = 0.68) and a trend towards an interaction with GN (F(1,26) = 3.001, p = 0.10). When males and females were analyzed separately, there were no significant differences between GS males and females (p = 1.0). There were no differences between GS and GN animals in males (p = 1.0) or females (0.766), however GN females had significantly more D2 binding compared to GN males (p=0.02).

D3 binding in the ventral striatum was significantly impacted by GN (F(1,22) = 7.108, p = 0.014) and by ventral striatal subregion (F(3,66) = 117.495, p<0.001), but not sex (F(1,22) = 0.447, p=0.51) nor the interaction of sex with GN (F(1,22) = 0.653, p=0.43). Given the significant effect of subregion, each was analyzed separately. GN effects were more robust in the rostral than caudal NAc core (Rostral NAc core: GN: F(1,23)=5.517, p=0.028; sex (F(1,23) = 0.290, p=0.60; interaction F(1,23) = 0.540, p = 0.47; Caudal NAc core: GN(1,23) = 3.257, p=0.08; sex: F(1,23) = 0.837, p = 0.37; interaction F(1,23) = 0.067, p = 0.80). In contrast, in the NAc shell showed more robust GN effects in the caudal compared to rostral segments (Caudal shell: GN: F(1,24)=8.452, p=0.008; sex: (F(1,24)=1.238, p=0.28; interaction: F(1,24)=0.305, p=0.59; Rostral shell GN: F(1,24)=2.299, p=0.14; sex: (F(1,24)=0.200, p=0.66; interaction: F(1,24)=0.001, p=0.98).

1. Matta SG, Balfour DJ, Benowitz NL, Boyd RT, Buccafusco JJ, Caggiula AR, et al. Guidelines on nicotine dose selection for in vivo research. Psychopharmacology (Berl). 2007;190(3):269-319. doi:10.1007/s00213-006-0441-0

2. Benowitz NL, Jacob P, 3rd. Daily intake of nicotine during cigarette smoking. Clinical pharmacology and therapeutics. 1984;35(4):499-504.

3. Franke RM, Belluzzi JD, Leslie FM. Gestational exposure to nicotine and monoamine oxidase inhibitors influences cocaine-induced locomotion in adolescent rats. Psychopharmacology. 2007;195(1):117-24. doi:10.1007/s00213-007-0876-y

4. Smith TD, Gallagher M, Leslie FM. Cholinergic binding sites in rat brain: analysis by age and cognitive status. Neurobiology of aging. 1995;16(2):161-73. doi:10.1016/0197-4580(94)00156-1

5. Kitchen I, Leslie FM, Kelly M, Barnes R, Crook TJ, Hill RG, et al. Development of delta-opioid receptor subtypes and the regulatory role of weaning: radioligand binding, autoradiography and in situ hybridization studies. The Journal of pharmacology and experimental therapeutics. 1995;275(3):1597-607.

6. Winzer-Serhan UH, Raymon HK, Broide RS, Chen Y, Leslie FM. Expression of alpha 2 adrenoceptors during rat brain development--II. Alpha 2C messenger RNA expression and [3H]rauwolscine binding. Neuroscience. 1997;76(1):261-72. doi:10.1016/s0306-4522(96)00369-7

7. Cao J, Lotfipour S, Loughlin SE, Leslie FM. Adolescent maturation of cocaine-sensitive neural mechanisms. Neuropsychopharmacology : official publication of the American College of Neuropsychopharmacology. 2007;32(11):2279-89. doi:10.1038/sj.npp.1301349

8. Franke RM, Park M, Belluzzi JD, Leslie FM. Prenatal nicotine exposure changes natural and drug-induced reinforcement in adolescent male rats. The European journal of neuroscience. 2008;27(11):2952-61. doi:10.1111/j.1460-9568.2008.06253.x

9. McQuown SC, Dao JM, Belluzzi JD, Leslie FM. Age-dependent effects of low-dose nicotine treatment on cocaine-induced behavioral plasticity in rats. Psychopharmacology. 2009;207(1):143-52. doi:10.1007/s00213-009-1642-0

10. Cao J, Belluzzi JD, Loughlin SE, Dao JM, Chen Y, Leslie FM. Locomotor and stress responses to nicotine differ in adolescent and adult rats. Pharmacology, biochemistry, and behavior. 2010;96(1):82-90. doi:10.1016/j.pbb.2010.04.010

11. Dao JM, McQuown SC, Loughlin SE, Belluzzi JD, Leslie FM. Nicotine alters limbic function in adolescent rat by a 5-HT1A receptor mechanism. Neuropsychopharmacology : official publication of the American College of Neuropsychopharmacology. 2011;36(7):1319-31. doi:10.1038/npp.2011.8

12. Marshall JF, Navarrete R, Joyce JN. Decreased striatal D1 binding density following mesotelencephalic 6-hydroxydopamine injections: an autoradiographic analysis. Brain research. 1989;493(2):247-57.

13. Harrod SB, Mactutus CF, Bennett K, Hasselrot U, Wu G, Welch M, et al. Sex differences and repeated intravenous nicotine: behavioral sensitization and dopamine receptors. Pharmacology, biochemistry, and behavior. 2004;78(3):581-92. doi:10.1016/j.pbb.2004.04.026
